# Changes in Acute Phase Response Biomarkers in Racing Endurance Horses

**DOI:** 10.3390/ani12212993

**Published:** 2022-10-31

**Authors:** Karla Mihelić, Zoran Vrbanac, Krunoslav Bojanić, Tara Kostanjšak, Blanka Beer Ljubić, Jelena Gotić, Dražen Vnuk, Nika Brkljača Bottegaro

**Affiliations:** 1 Faculty of Veterinary Medicine, University of Zagreb, Heinzelova 55, 10 000 Zagreb, Croatia; 2 Department of Radiology, Ultrasound Diagnostic and Physical Therapy, Faculty of Veterinary Medicine, University of Zagreb, Heinzelova 55, 10 000 Zagreb, Croatia; 3 Laboratory for Aquaculture Biotechnology, Division of Materials Chemistry, Ruđer Bošković Institute, Bijenička Cesta 54, 10 000 Zagreb, Croatia; 4 Clinic for Surgery, Orthopaedics and Ophthalmology, Faculty of Veterinary Medicine, University of Zagreb, Heinzelova 55, 10 000 Zagreb, Croatia; 5 Clinic for Internal Diseases, Faculty of Veterinary Medicine, University of Zagreb, Heinzelova 55, 10 000 Zagreb, Croatia

**Keywords:** equine sport, acute phase biomarkers, acute phase response, calprotectin, endurance race

## Abstract

**Simple Summary:**

Sport horses competing in exhausting sporting events require special attention to ensure their welfare. Changes in acute phase response induced by both aerobic and anaerobic exercise are studied for understanding the physiological response to strenuous exercise. We suggest that endurance racing induces acute phase response in horses, characterized by a decrease in calprotectin and haptoglobin values, and an increase in concentrations of ceruloplasmin and albumin, with more pronounced changes noted at races with higher average speeds, suggesting the need for thorough horse monitoring during exhausting races.

**Abstract:**

This study aimed to evaluate if exercise-induced acute phase response (APR) occurs in endurance horses in response to the race. The study included 23 horses competing in an endurance competition with a successfully passed clinical examination before the race. Blood samples were collected before the start and within 30 min after the end of the race. Haematological and biochemical tests were performed and correlated to acute phase biomarkers changes. Values of calprotectin and haptoglobin (Hp) decreased after the races compared to values before, while concentrations of ceruloplasmin and albumin recorded a significant increase. Greater changes in calprotectin values were noted in Arabian horses compared to other breeds. Values of Hp showed a significantly greater decrease after longer races. Based on study results, endurance racing induces APR in horses characterised by significant changes in selected acute phase biomarkers. More pronounced changes were noted at races with higher average speeds, suggesting the need for thorough horse monitoring during exhausting races.

## 1. Introduction

Participation of athletic horses in exhausting sporting events requires an understanding of their physiological responses to ensure their welfare. Physiological, haematological and biochemical changes associated with exercise have been studied in horses of various equestrian disciplines such as show jumping [1,2], eventing [3,4], races [5,6] and endurance courses [7].

An increase in the levels of proinflammatory cytokines and acute phase proteins (APPs) is part of the characteristic acute phase response (APR) that occurs in inflammation [8]. The production of APPs is triggered by inflammatory mediators, interleukin 1β, interleukin 6 and tumour necrosis factor-α [9]. Haptoglobin (Hp), C-reactive protein (CRP) and serum amyloid A (SAA) are the most strongly reacting APPs in humans and animals [10] with significantly varying APP production among different species [11]. In equine practice, the most commonly assessed APPs are SAA, Hp and fibrinogen, whose levels vary concerning triggering events [12]. Concentrations of SAA increase rapidly and substantially within 24 h in response to inflammation or infectious conditions, including viral and bacterial respiratory infections, colic, surgery and experimentally induced septic arthritis [13,14]. Haptoglobin may be helpful to screen for and monitor intravascular haemolytic anaemia while its concentration increases in the presence of inflammatory diseases [15]. Plasma fibrinogen levels are used as an index of inflammation in the baseline hematologic evaluation of horses before anaesthesia [16].

Changes in APR can occur induced by an aerobic or anaerobic type of exercise [17] but the literature data about the effects of exercise on APR in horses are limited. The production of APPs, with an emphasis on SAA or Hp, has already been described in response to strenuous endurance races [18]. The APR in short-term strenuous exercises has been described in Thoroughbred racehorses analysing the concentration of SAA [19]. The start of the APR in jumping horses was reported after the end of the exercise with an influence on concentration levels of SAA [20,21], fibrinogen, and iron (Fe) [21].

The aim of this study was to determine if exercise-induced APR, defined by changes in selected acute phase biomarkers (calprotectin, Hp, ceruloplasmin (Cp), albumin and Fe) occurs in endurance horses in response to racing. The further aim was to find a potential correlation between changes in haematological, biochemical and APR parameters. We hypothesized that exercise-induced APR, characterized by changes in acute phase biomarkers, occurs in endurance horses in response to racing, with more pronounced changes during longer races and higher average speed. 

## 2. Materials and Methods

### 2.1. Competition and Conditions

The study included horses competing at one national competition held in April 2021 in Croatia. The horse owners were offered to participate in the study before the first veterinary inspection of their horse(s) at the competition sites. Three races held at the competition differed according to distances of 43, 67 and 86 km. Races of 67 and 86 km distances were classified as long while the race of 43 km as short.

### 2.2. Horses

All horses competing at the mentioned competition were eligible for enrolment in this study, while 23 horses were enrolled following the owner’s consent. Horses had been transported from varying distances and were given at least two hours of rest before the first veterinary examination. Horses were subjected to different diets and protocols of electrolyte supplementation, both during training and competition.

Horses were divided into two age groups: aged 10 years or under and older than 10 years. Horses were also divided by gender into a group of males (including stallions and geldings) and females. Concerning the breed, horses were divided into two groups: horses of the Arabian breed (Arabian breed and Arabian crossbred horses), and horses of other warmblood breeds. The average speed of horses in the study population was also evaluated with a mean of 15.49 km/h.

### 2.3. Sample Collection and Analysis

Blood samples (10 mL) were collected by jugular venepuncture into vacuum tubes for haematological and biochemical analysis using a vacutainer (Vacutainer^®^, Becton Dickenson, East Rutherford, NJ, USA). Horses were sampled twice, the first sample 1 h before the start and the second within 30 min after the end of the race. The blood samples for haematological analysis were shipped in cooling bags at 4 °C to the laboratory and analysis was performed immediately, while blood samples for biochemical analysis were left to coagulate for 30 min and thereafter centrifuged at 3500× *g* rpm for 15 min. The serum samples were shipped in cooling bags at 4 °C to the laboratory and stored at −20 °C until further analyses.

The haematological analysis was performed using a Horiba Scil Vet ABC Plus analyser (Scil, Viernheim, Germany). The differential leukocyte count was obtained from blood smears stained according to the Pappenheim method and examined under an Olympus BX41 microscope (Olympus, Tokyo, Japan). The concentrations of total proteins, albumin, urea, cholesterol, triglycerides, Fe and the enzyme activities of aspartate aminotransferase (AST), gamma-glutamyl transferase (GGT), creatine phosphokinase (CPK) and lactate dehydrogenase (LDH) were analysed by Abbott commercial kits using an automated biochemistry analyser Architect c4000 (Abbott, Abbott Park, IL, USA). The concentration of Hp was determined by an automated spectrophotometric haemoglobin-HP binding assay [22,23]. The Cp concentration was determined by an automated spectrophotometric assay using a biochemistry analyser Architect c4000 (Abbott, USA) [24]. Serum calprotectin concentrations were measured using a Horse Calprotectin ELISA Kit (MyBioSource, San Diego, CA, USA) according to the manufacturer’s instructions. Calprotectin assay was performed using only one kit and thus, just the intra-essay coefficient of variation was reported. The average intra-assay CV of calprotectin assay was 7.8%. For Hp, Cp, albumin and Fe the average intra-assay CVs were 2.75%; 1.7%; 0.8%; and 1.15%, and inter-assay CVs were 5.46%; 2.94%; 1.3%; and 0.85%, respectively.

Reference ranges were taken from the equine reference intervals used by the Laboratory of Veterinary Internal Medicine, Faculty of Veterinary Medicine, Zagreb, where the samples were analysed.

### 2.4. Statistical Methods

Statistical and exploratory data analyses were performed using R v4.0.4 (R: A language and environment for statistical computing. R Core Team (2021). R Foundation for Statistical Computing, Vienna, Austria. URL http://www.R-project.org/ (accessed on 5 October 2021)). Exploratory analyses included standard measures of central tendency and dispersion. The normality of data was evaluated using the Shapiro–Wilks test. Normally distributed data were analysed using the paired Student’s *t*-test with an application of Welch’s correction in cases of significant heterogeneity of variance according to the F test of homogeneity of variance. Non-normally distributed data were analysed using the paired Wilcoxon signed-rank test. Delta change of haematological and biochemical data was calculated by subtracting values before the race from values after the race. The association of delta values with age, gender, breed, distance and speed were also analysed using Student’s *t*-test and the Mann–Whitney test according to a normal distribution of data. Categorical data were explored using counts and proportions and analysed using chi-square and Fisher’s test as indicated by the frequency of cases in contingency tables. For correlation analyses of delta values, Spearman’s correlation tests were used. All tests used an alpha error set at <0.05.

## 3. Results

All the horses participating in the study successfully passed the veterinary inspection before the start and completed the required race distance. Out of twenty-three horses enrolled in the study, there were 12 female and 11 male horses while 12 horses out of 23 enrolled were aged 10 years or under. There were 10 Arabian horses and 13 horses of other warmblood breeds. Concerning race distance, there were 12 race starts (seven females and five males) at the short distance with an average speed of 15.45 (±1.34 SD) km/h and 11 race starts (five females and six males) at the long distance with an average speed of 15.40 (±0.77 SD) km/h. At each race distance, six horses aged 10 years or younger participated. The horses were competing in numerous different race categories according to the age of the riders. Considering the low number of horses per individual class, the ranking position could not have been analysed and was not reported.

The overall values of acute phase biomarkers in horses before and after competing in endurance races are presented in Table 1. While values of calprotectin and Hp after the races showed a significant decrease compared to values before, a significant increase in Cp and albumin was noted. The concentration of Fe after the race did not change compared to the concentration before.

The overall values of haematological and biochemical parameters are shown in Table 2 and Table 3. While changes in values after the races compared to values before the races for haematological parameters greatly differed between parameters, a significant increase in all biochemical parameters except globulins was noted.

**Figure 1:**
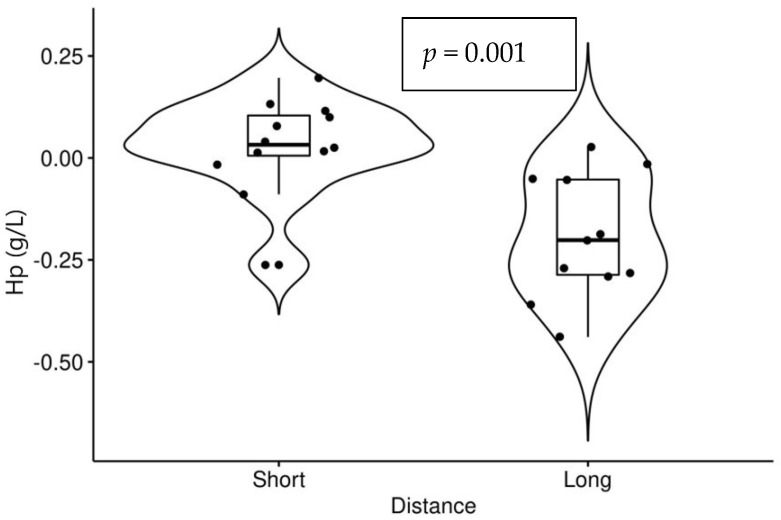
Changes in values of haptoglobin (Hp) (after races–before races) in short (12 horses) and long (11 horses) race distances.

Significant changes were noted after the races compared to values before also among different categories. Values of Hp showed a significantly greater decrease after the longer race (*p* < 0.001) (Figure 1).

**Figure 2:**
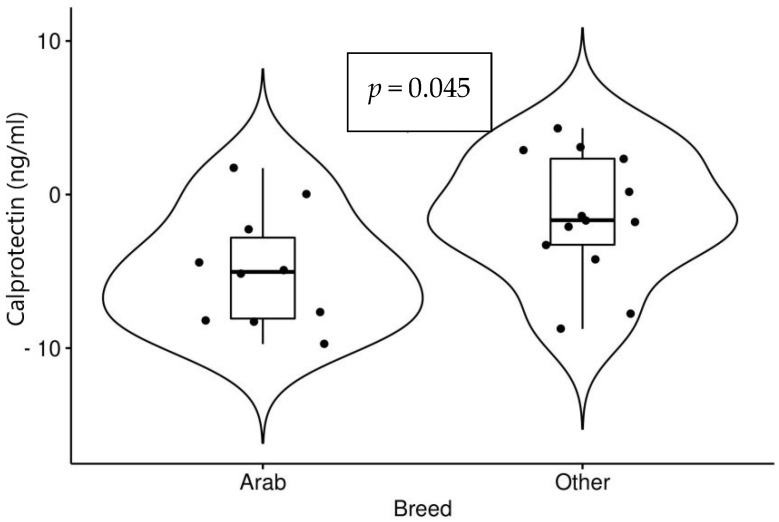
Changes in values of calprotectin (after races−before races) in 10 Arabian horses and 13 horses of other breeds.

**Figure 3:**
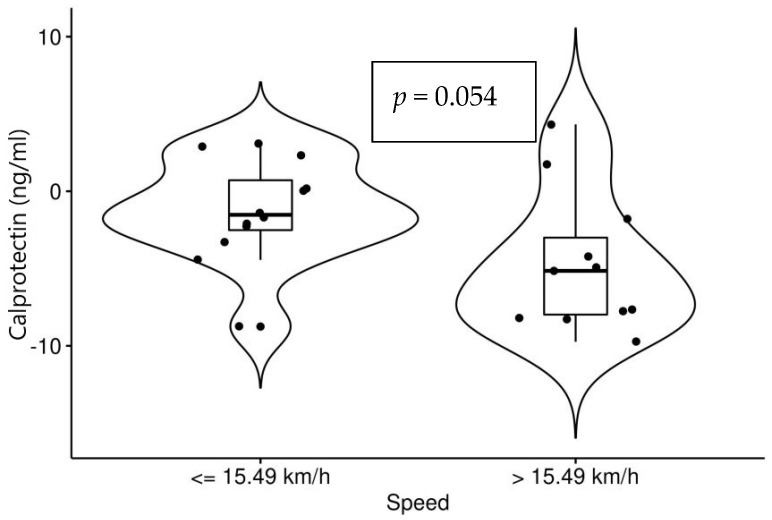
Changes in values of calprotectin (after races–before races) in horses of slower (12 horses) and higher speed (11 horses).

A significant decrease in values of calprotectin after the races was noted among all horses. Arabian horses showed a more significant decrease compared to other breeds (*p* = 0.045). Horses with higher speeds recorded a greater decrease compared to horses with slower speeds which, although not significant (*p* = 0.054), is considered clinically relevant. Both are presented in Figure 2 and Figure 3.

All the horses showed a significant increase in LDH, AST, CPK, triglycerides and urea after the races. With regard to distance, longer races resulted in a significantly greater increase in LDH (*p* < 0.001), AST (*p* = 0.003), CPK (*p* = 0.001), triglycerides (*p* = 0.016) and urea (*p* = 0.002).

**Figure 4:**
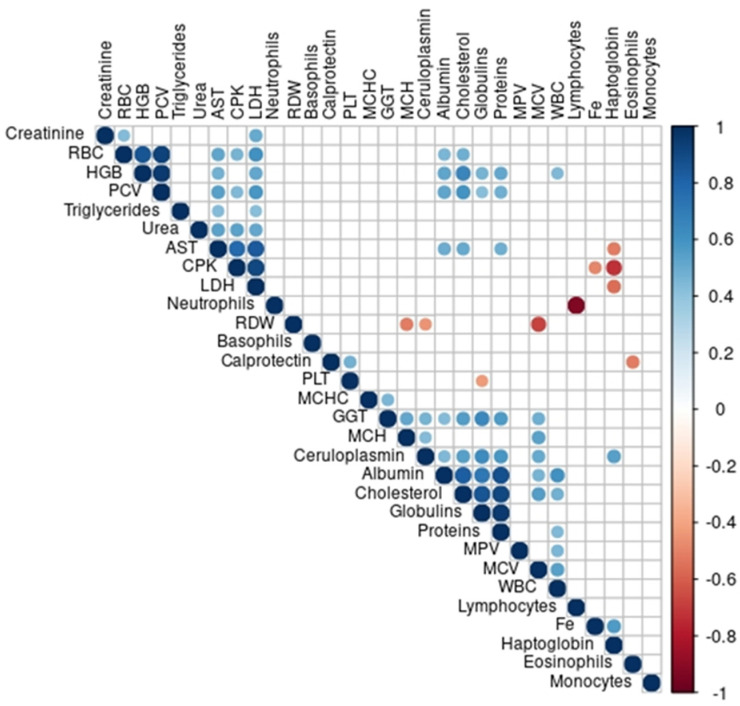
Correlations of changes in acute phase biomarkers, and haematological and biochemical parameters in 23 horses, shown by correlogram. Only statistically significant correlations (*p* < 0.05) were shown using a correlation coefficient ranging from −1 to 1, in colour, where blue indicates linear positive correlations, while red indicates negative linear correlations. The strength of the correlation increases with an increase in the size of the circle and the intensity of the colour. Abbreviations: AST—aspartate aminotransferase; CPK—creatine phosphokinase; Fe—iron; GGT—gamma-glutamyl transferase; HGB—haemoglobin; LDH—lactate dehydrogenase; MCH—mean corpuscular haemoglobin; MCHC—mean corpuscular haemoglobin concentration; MCV—mean corpuscular volume; MPV—mean platelet volume; PCV—packed cell volume; PLT—platelets; RBC—red blood cell; RDW—red cell distribution width; WBC—white blood cell.

Correlations of changes in acute phase biomarkers and haematological and biochemical parameters are presented by correlogram (Figure 4). A positive correlation was found between changes in the concentrations of calprotectin and platelets, while Cp positively correlated with values of cholesterol. The concentration of Hp showed a negative correlation in comparison to concentrations of AST, CPK and LDH, and a positive correlation with Fe and Cp.

## 4. Discussion

The results of the present study showed that endurance race-induced APR in horses is characterized by changes in serum values of observed acute phase biomarkers including calprotectin, Hp, Cp and albumin.

Values of serum calprotectin showed a significant decrease after the races. To date, to the best of our knowledge, no study has been published on calprotectin in horses in response to exercise. The evaluation of calprotectin expression along with an assessment of epithelial and endothelial apoptosis in the colon was performed in horses with laminitis [25], while serum calprotectin was measured in equine colon ischemia [26]. In dogs, a non-invasive assessment of faecal stress biomarkers in hunting dogs showed no differences in calprotectin gene expression during exercise and at rest [27]. The responses in serum levels of novel inflammatory proteins, including calprotectin, were studied in 12 physically active people before and after completing various types of extreme physical exertion which induced an increase in serum calprotectin values that returned to baseline within 48 h [28]. The studies concluded that calprotectin release may indicate exercise-induced activation of neutrophil-driven inflammation, which has previously been linked with anti-inflammatory processes and scavenging of tissue-damaging reactive oxygen species. Post-exercise increases in serum calprotectin also coincided with increased leukocyte counts, suggesting that calprotectin could also serve as a biomarker for myeloid reactions when analysing the body’s immune response to exercise. In our study, a significant increase in leukocytes and neutrophils was noted with a decrease in calprotectin values. The main difference, regarding blood sample collection, between the study in humans [28] and this study is the time of blood collection, which in their study was on the day before the race and then 3 and 48 h post-exercise, while in this study blood was collected an hour before the race start and immediately after race completion. Another study reported that calprotectin from sweat in humans can be used as a real-time indicator for inflammatory bowel disease (IBD) flare-ups [29]. Basal calprotectin sweat concentration measured in healthy patients was 379 ng/L while the basal concentration of sweat calprotectin in patients with IBD amounted to 620 ng/L. These results prove the presence of calprotectin in sweat and, if we assume that calprotectin is present in horse sweat as well, it could explain the decrease of calprotectin concentrations in horses after vigorous physical activity. It has been reported that to maintain homeostasis, the individual horse produces 10–15 litres of sweat per hour at an average speed of 16 km/h [30]. In our study, the average speed was 15.49 km/h and all horses were dehydrated after the race, probably due to sweat loss, as demonstrated by the increased PCV and albumin concentrations. If calprotectin is indeed excreted via sweat in horses, this could explain its reduction in the immediate post-race period. The result of our research, in which horses with higher speed and therefore more sweat production recorded a greater decrease in calprotectin values compared to horses with slower speed, although not statistically significant, is in support of this thesis. To our knowledge, there is no known research about calprotectin in equine sweat or that of other animals.

Due to the low molecular weight (36.5 kDa) of calprotectin in humans, its half-life is only 5 h once it diffuses into circulation [31,32]. In our study, the second blood collection was approximately more than 5 h after the short race started and more than 7 h after the long race started. The data for the half-life of calprotectin in horses is not available; it can be assumed that the results obtained in this study are also attributable to the calprotectin breakdown before the second sampling.

A significant decrease in values of calprotectin after the races was noted among all horses but the Arabian horses showed a more significant decrease compared to other breeds, which can be explained by breed differences. To date, to the best of our knowledge, no study has been published on calprotectin in different equine breeds.

In this study, a positive correlation was found between changes in the concentrations of calprotectin and platelets. Calprotectin has been shown to induce a thrombogenic, inflammatory response in endothelial cells in humans by increasing the transcription of pro-inflammatory chemokines and adhesion molecules [33], which may secondarily activate platelets. However, a direct effect of calprotectin on platelet aggregation has not yet been established.

The values of Hp significantly decreased after the races. Haptoglobin was identified as a haemoglobin-binding protein and the principal scavenger of free haemoglobin in the blood [34]. A decrease in Hp concentration after exertion has been described in horses as a result of intravascular haemolysis [35]. Exercise-induced intravascular haemolysis can result from increased fragility of erythrocytes because of frequent accumulation in the spleen, strong shear forces associated with haemoconcentration and increased blood flow, as well as oxidative stress and anaerobic exercise leading to a decrease in blood pH, increase in partial pressure of carbon dioxide and increase in blood lactate concentration [36,37]. Since the values of haemoglobin significantly increased after the race, it may be concluded that the level of Hp decreased due to the binding of free haemoglobin released from damaged erythrocytes. In a study [38] horses were subjected to two different tests on a treadmill, one consisting of short-duration and rapid-acceleration mostly anaerobic training and the other of long-duration and slow-acceleration predominantly aerobic training. A significant decrease in Hp was noticed only during short-duration and rapid-acceleration training. In our study, although overall values of Hp decreased after the race, a more significant decrease was noticed after a long race. It is to be expected that endurance races have a much greater impact on metabolic changes than treadmill exercise in horses, including changes in APP, which is why Hp decreased due to intravascular haemolysis in short and long races, with more pronounced changes in longer races. A negative correlation was found between changes in the concentration of Hp in comparison to concentrations of AST, CPK and LDH. Increased values of AST, CPK and LDH indicate muscle damage accompanied by haemolysis, which requires the consumption of Hp leading to its decreased values.

In the present study, a positive correlation between changes in Hp and both Fe and Cp was noted. Haemolysis during exercise results in the release of a larger amount of Fe. Copper, as part of a copper-containing plasma protein (Cp), plays an important role in transmembrane iron transport and transfer of iron from ferritin (ferrous form) to transferrin (ferric form) [39] which explains the positive correlation noted in this study. A significant increase in Cp after the race noted in this study is consistent with another study’s result [8]. Carvalho Filho et al. [8] evaluated the serum of 10 horses in a show-jumping competition and Cp showed a progressive increase in concentrations between values before the competition and all measurements following the completion of the competition. The increase in Cp in our study is related to the increase in oxygen-free radicals that are produced when there is an inflammatory process or stress condition caused by the physical activity that the horses are submitted to [8,40]. The concentration of Cp was positively correlated with values of cholesterol. In our study, the concentration of cholesterol significantly increased after the race, which is consistent with the other studies explaining that the increase in the concentration of cholesterol may be a consequence of lipid mobilization due to intensive physical activity [41,42]. Based on the collected data, the authors of [43] suggested that Cp inhibits superoxide and ferritin-dependent lipid peroxidation via its ability to incorporate reductively mobilized iron into ferritin. As Cp concentration significantly increased in horses after the race, it may be concluded this was to inhibit lipid peroxidation.

The concentration of albumin significantly increased after the race, which is similar to other study results [38] where albumin increased in horses submitted to long-duration and slow-acceleration training. During exercise in horses, fluid is displaced from the intravascular space to the interstitial and intracellular space, which leads to an increase in albumin concentrations and is related to the length and intensity of the type of exercise [17]. As horses in our study were participating in endurance races with the shortest race distance of 43 km, and horses in another study [38] participated in a long-duration exercise, it could be considered that profuse sweating during longer race distances could be markedly causing the fluid shift and increase in albumin concentrations. The fact that albumin is considered a negative APP in horses [44] whose values are decreased in APR due to down-regulation of hepatic albumin synthesis in APR [45], allows the conclusion that in our study the horses were subjected to a more significant effect of dehydration and sweating than the acute phase response.

During long races, horses are exposed to thermolysis, electrolyte loss and the emergence of large amounts of catabolism products as a consequence of haemolysis, partial rhabdomyolysis, energy metabolism, and liver and kidney metabolism. All of the above contribute to changes in haematological and biochemical parameters noted in this study through an increased RBC count, stress neutrophilia and lymphopenia, increase in musculoskeletal parameters CPK, LDH and AST, increase in TP, GGT, urea, creatinine, triglycerides and cholesterol. Values of studied haematological and biochemical parameters are consistent with the results of previous research in endurance horses [7,42].

This present study contains certain limitations that need to be emphasized. Enrolment in the study was voluntary and it did not include all the participating horses. This represents a possible selection bias that is extremely difficult to avoid in cross-sectional field studies. A relatively small number of horses per evaluated category could have also influenced the results. Another limitation was a lack of standardized feeding or detailed nutritional surveys, although most of the horses were fed balanced formulas for endurance horses. Since all the horses had been transported to the event from different locations, the transport per se could have also influenced the results despite all the horses having been given a period of rest in proportion to the distance and stress caused by the transportation before the start of the competition. However, it was considered that the transportation effect had ceased since all the animals had successfully passed the veterinary examination and were declared fit to compete before the blood sampling. Another limitation was the lack of calprotectin measurement in horse sweat which could support our thesis of calprotectin reduction due to sweat excretion. Further studies are needed to investigate the variation in parameters, sampling time influence and to determine the time for recovery to basal values, to define the possible effects on the metabolism of the athlete horse.

## 5. Conclusions

Endurance races induced APR in horses and caused significant changes in values of observed acute phase biomarkers, including calprotectin, Hp, Cp and albumin. The acute phase response was characterised by a decrease in calprotectin and Hp values and an increase in concentrations of Cp and albumin. The observed changes were also noted during shorter races emphasizing the need for close monitoring even of horses competing at lower-level rides. The availability of veterinary professionals should be mandatory at all endurance events regardless of the race distance.

## Figures and Tables

**Table 1:** Values of serum acute phase biomarkers in 23 horses before and after the endurance race.

Parameter	Reference Values	Pre-Race Value	Post-Race Value	Statistical Significance
Calprotectin (ng/mL)	-	30.08 ± 7.77	27.17 ± 8.36	**10% ↓ *p* = 0.003**
Hp (g/L)	-	0.62 ± 0.18	0.55 ± 0.27	**11% ↓ *p* = 0.045**
Cp (g/L)	-	0.28 ± 0.05	0.33 ± 0.06	**18% ↑ *p* < 0.001**
Albumin (g/L)	26–37	34.74 ± 2.9	38.22 ± 3.18	**10% ↑ *p* < 0.001**
Fe (μmol/L)	17–38.8	24.83 ± 7.96	25.68 ± 8.92	NS

Abbreviations: Cp—ceruloplasmin; Hp—haptoglobin; NS—not significant. % = [(mean post-race value − mean pre-race value)/(mean prevalue) ∗ 100]. Reference values are given by the analysing laboratory. Data represent the mean ± standard deviation.

**Table 2:** Values of haematological parameters in 23 horses before and after the endurance race.

Parameter	Reference Values	Pre-Race Value	Post-Race Value	Statistical Significance
RBC (×10^12^/L)	6–12	8.46 ± 1.19	9.91 ± 1.01	**17% ↑ *p* < 0.001**
HGB (g/L)	100–180	115.43 ± 14.8	133.74 ± 14.41	**16% ↑ *p* < 0.001**
PCV (%)	32–48	37.39 ± 4.73	43.74 ± 4.73	**17% ↑ *p* < 0.001**
MCV (fL)	34–58	44.3 ± 2.55	44.04 ± 1.94	NS
MCH (pg)	13–19	13.83 ± 0.98	13.65 ± 0.71	NS
MCHC (g/L)	310–370	309.7 ± 6.46	306.43 ± 2.66	**1% ↓ *p* = 0.04**
RDW (%)	-	17.48 ± 0.85	17.65 ± 0.49	NS
PLT (×10^9^/L)	100–600	292.13 ± 112.55	293.96 ± 141.53	NS
MPV (fL)	-	7.04 ± 0.56	6.96 ± 0.56	NS
WBC (×10^9^/L)	6–12	9.19 ± 1.89	11.49 ± 2.77	**25% ↑ *p* < 0.001**
Neutrophils (%)	67–	64.22 ± 11.9	81.22 ± 7.82	**26% ↑ *p* < 0.001**
Lymphocytes (%)	25–60	31.13 ± 12.2	17.35 ± 7.52	**79% ↓ *p* < 0.001**
Monocytes (%)	1–8	0.39 ± 0.94	0.52 ± 0.85	NS
Eosinophils (%)	3–	3.26 ± 3.53	0.74 ± 1.36	**77% ↓ *p* = 0.002**
Basophils (%)	-	0.48 ± 1.04	0.17 ± 0.58	NS

Abbreviations: HGB—haemoglobin; MCH—mean corpuscular haemoglobin; MCHC—mean corpuscular haemoglobin concentration; MCV—mean corpuscular volume; MPV—mean platelet volume; NS—not significant; PCV—packed cell volume; PLT—platelets; RBC—red blood cell; RDW—red cell distribution width; WBC—white blood cell. % = [(mean post-race value − mean pre-race value)/(mean prevalue) ∗ 100]. Reference values are given by the analysing laboratory. Data represent the mean ± standard deviation.

**Table 3:** Values of serum biochemical parameters in 23 horses before and after the endurance race.

Parameter	Reference Values	Pre-Race Value	Post-Race Value	Statistical Significance
Globulins (g/L)	-	33.83 ± 6.25	35.04 ± 10.63	NS
Total proteins (g/L)	55–75	68.6 ± 5.67	74.6 ± 5.59	**9% ↑ *p* < 0.001**
GGT (U/L)	0–28	16.65 ± 6.1	18.17 ± 6.56	**9% ↑ *p* < 0.001**
AST (U/L)	0–490	294.17 ± 41.71	365.57 ± 55.36	**24% ↑ *p* < 0.001**
LDH (U/L)	162–412	326.52 ± 73.94	504.61 ± 112.37	**54% ↑ *p* < 0.001**
CPK (U/L)	0–130	219.3 ± 71.38	787.7 ± 578.6	**259% ↑ *p* < 0.001**
Urea (mmol/L)	3.3—6.6	4.77 ± 0.81	6.78 ± 1.11	**42% ↑ *p* < 0.001**
Creatinine (U/L)	0–115	81.83 ± 10.38	111.9 ± 16.53	**37% ↑ *p* < 0.001**
Triglycerides (mmol/L)	0.1–0.5	0.217 ± 0.08	0.29 ± 0.12	**34% ↑ *p* = 0.01**
Cholesterol (mmol/L)	1.8–4.6	2.15 ± 0.37	2.31 ± 0.42	**7% ↑ *p* < 0.001**

Abbreviations: AST—aspartate aminotransferase; CPK—creatine phosphokinase; Fe—Iron; GGT—gamma-glutamyl transferase; LDH—lactate dehydrogenase; NS—not significant. % = [(mean post-race value − mean pre-race value)/(mean prevalue) ∗ 100]. Reference values are given by the analysing laboratory. Data represent the mean ± standard deviation.

## Data Availability

The datasets used and/or analysed for the present study are available from the corresponding author upon reasonable request.
